# Diabetes and Obesity Modify the Effect of Alcohol Consumption on Carbohydrate‐Deficient Transferrin

**DOI:** 10.1002/edm2.70112

**Published:** 2025-10-23

**Authors:** Tomás González‐Vidal, Óscar Lado‐Baleato, Fátima de la Osa, Manuela Alonso‐Sampedro, Carmen Fernández‐Merino, Juan Sánchez‐Castro, Francisco Gude, Arturo González‐Quintela

**Affiliations:** ^1^ Department of Endocrinology and Nutrition Hospital Universitario Central de Asturias/University of Oviedo Oviedo Spain; ^2^ Instituto de Investigación Sanitaria del Principado de Asturias (ISPA) Oviedo Spain; ^3^ Department of Medicine University of Oviedo Oviedo Spain; ^4^ Research Methods Group (RESMET), Health Research Institute of Santiago de Compostela (IDIS), Network for Research on Chronicity, Primary Care, and Health Promotion (RICAPPS‐ISCIII) Santiago de Compostela Spain; ^5^ ISCIII Support Platforms for Clinical Research, Health Research Institute of Santiago de Compostela Santiago de Compostela Spain; ^6^ Department of Medicine University of Santiago de Compostela Santiago de Compostela Spain; ^7^ Department of Primary Care A Estrada Primary Care Centre A Estrada Spain; ^8^ Department of Primary Care Concepción Arenal Primary Care Centre Santiago de Compostela Spain; ^9^ Department of Internal Medicine Complejo Hospitalario Universitario de Santiago de Compostela Santiago de Compostela Spain

**Keywords:** alcohol, carbohydrate‐deficient transferrin, diabetes, insulin resistance, obesity, sialylation

## Abstract

**Aims:**

Serum levels of carbohydrate‐deficient transferrin (CDT, the sum of its asialylated and disialylated glycoforms) are a commercial marker of alcohol abuse. Our aim was to investigate the potential influence of metabolic factors on serum CDT levels and the predictive value of transferrin glycoforms for the development of type 2 diabetes in a general adult population.

**Materials and Methods:**

We measured serum CDT levels by capillary electrophoresis in 1516 individuals (median age 52 years; 55.3% women) randomly selected from the general adult population of a municipality.

**Results:**

Insulin resistance and the associated body mass index and diabetes modified the effect of alcohol consumption on CDT levels; i.e., CDT in heavy drinkers was lower in individuals with obesity than in lean counterparts and was also lower in people with diabetes than in normoglycaemic individuals. The relative abundance of transferrin glycoforms was not significantly associated with the development of type 2 diabetes after a mean follow‐up of 7.4 years.

**Conclusions:**

There is an interaction between alcohol consumption and factors associated with insulin resistance in relation to transferrin sialylation. The sensitivity of CDT for detecting heavy alcohol consumption might be limited in people with obesity or diabetes.

## Graphical abstract

There is an interaction between alcohol consumption and factors associated with insulin resistance in relation to transferrin sialylation. The sensitivity of carbohydrate‐deficient transferrin for detecting heavy alcohol consumption might be limited in patients with obesity or diabetes.

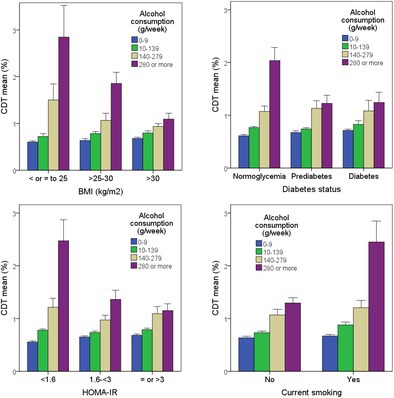

## 1 Introduction

Glycosylation is the most common co‐ and post‐translational modification of proteins in mammals [1, 2, 3, 4]. It plays an essential role in the folding, stability, solubility, and effector function of proteins. Glycans attached to glycoproteins and glycolipids are therefore involved in virtually all biological processes and in the pathophysiology of every disease; glycosylation changes can therefore serve as markers for diverse diseases [1, 2, 3, 4]. Sialic (*N*‐acetylneuraminic) acids are negatively charged amino sugars commonly found as terminal residues in glycans, where they are involved in many molecular and cellular interactions [1, 5, 6]. Transferrin is a glycoprotein produced in the liver that is normally present in human plasma with the function of transporting iron [7]. It has several isoforms resulting from differences in its glycosylation [7, 8, 9, 10]. Transferrin has two *N*‐glycosylation sites at asparagine residues 432 and 630 [7, 11]. Transferrin *N‐*glycans are predominantly diantennary and triantennary sialylated complex‐type glycans, with or without fucose, with afucosylated diantennary disialylated glycans being the most abundant [7, 11, 12]. Thus, under normal conditions, approximately 15% of transferrin circulates as pentasialotransferrin, 75% as tetrasialotransferrin, 5% as trisialotransferrin, less than 2% as disialotransferrin, and less than 1% as monosialo‐ or asialotransferrin [9]. The term carbohydrate‐deficient transferrin (CDT) refers collectively to the group of transferrin glycoforms with a lower degree of glycosylation; specifically, a lack of sialic acid residues, that is, the sum of disialo‐, monosialo‐ (almost absent under normal conditions) and asialotransferrin [8, 9, 10]. CDT can be calculated by various methods [9, 13], including commercially available capillary electrophoresis [14].

Given that alcohol consumption has been associated with protein glycosylation disorders [15], CDT increase has become a well‐known marker of alcohol abuse [8, 9, 10, 16, 17, 18, 19]. The mechanism for CDT increase in alcohol abusers remains unclear but may involve an alteration in the activity of glycosyltransferases induced by acetaldehyde, the main metabolite of ethanol [20, 21, 22]. Metabolic disorders can also affect CDT levels, although this has not been well studied. Previous studies have found associations and/or interactions between CDT and the body mass index (BMI) [16, 17, 23, 24, 25], suggesting that CDT might be a poor marker of excessive alcohol consumption in people with overweight or obesity [26, 27]. Although the authors did not measure insulin concentrations, they speculated that insulin resistance is associated with reduced CDT sensitivity to alcohol intake [26, 27]. In fact, another study showed that people with insulin resistance (as determined by hyperinsulinemic euglycaemic clamp studies) were less likely to have high CDT concentrations [28].

To our knowledge, no population‐based studies have investigated the association of CDT with insulin resistance, diabetes and other components of metabolic syndrome. The aim of this study was to investigate the possible association of CDT with these metabolic variables and to explore the predictive value of transferrin sialylation for the development of type 2 diabetes mellitus (T2DM).

## 2 Materials and Methods

### 2.1 Study Design and Setting

A detailed description of this cohort study (A‐Estrada Glycation and Inflammation Study [AEGIS], NCT01796184; www.clinicaltrials.gov) has been published elsewhere [29]. The study was conducted in the municipality of A‐Estrada, in northwestern Spain. An age‐stratified random sample of the population aged ≥ 18 years was drawn from the Spanish National Health System Registry, which covers more than 95% of the Spanish population. Between November 2012 and March 2015, participants were consecutively invited to the Primary Care Centre to complete an interviewer‐administered, structured questionnaire that collected demographic, anthropometric, and lifestyle information. They also provided a fasting venous blood sample. The study population consisted of 1516 participants (838 [55.3%] women; median age 52 [range 18–91 years]).

### 2.2 Main Determinations

#### 2.2.1 Lifestyle Variables

Alcohol consumption. It was measured in standard drinking units by summing the number of glasses of wine (approximately 10 g of alcohol), bottles of beer (approximately 10 g of alcohol), and units of spirits (approximately 20 g alcohol) regularly consumed per week [15, 30]. All participants also completed the Alcohol Use Disorders Identification Test (AUDIT) questionnaire, which has been validated in Spain [31].

Smoking. It was recorded as the number of cigarettes regularly consumed per day. Consumers of ≥ 1 cigarette per day were considered smokers. Individuals who had quit smoking during the preceding year were considered smokers.

Physical activity. All participants completed the International Physical Activity Questionnaire (short form) and the metabolic equivalents of task were subsequently calculated [32].

#### 2.2.2 Metabolic Determinations

Body mass index. Participants were classified as having normal weight (≤ 25 kg/m^2^), overweight (> 25–30 kg/m^2^), or obesity (> 30 kg/m^2^).

Metabolic syndrome. Participants were considered to have metabolic syndrome if they met ≥ 3 of the Adult Treatment Panel III criteria: (1) abdominal obesity (waist circumference > 102 cm for men and > 88 cm for women); (2) hypertriglyceridemia (fasting serum triglycerides ≥ 150 mg/dL); (3) low high‐density lipoprotein (HDL) cholesterol levels (fasting HDL‐cholesterol levels < 40 mg/dL for men and < 50 mg/dL for women); (4) elevated blood pressure (arterial blood pressure ≥ 130/≥ 85 mmHg or current use of antihypertensive medication; for those on antihypertensive medications, the specific antihypertensive drug class was also recorded); and (5) hyperglycaemia (fasting serum glucose levels ≥ 110 mg/dL or current use of antidiabetic therapy; for those on antidiabetic medications, the specific antidiabetic drug class was also recorded) [33].

Glycaemic status. Individuals were considered to have diabetes if they had been previously diagnosed with diabetes or had a glycated haemoglobin (HbA1c) level ≥ 6.5% and/or a fasting plasma glucose concentration ≥ 126 mg/dL [34]. Individuals with prediabetes were defined as those with an HbA1c between 5.7% and 6.4% or a fasting plasma glucose between 100 and 125 mg/dL [34]. The remainder were considered normoglycaemic [34]. Individuals with diabetes (*n* = 183) were further classified according to diabetes type (178 [97.2%] participants had T2DM [among whom hyperglycaemia was considered to have been induced by glucocorticoids in 2 individuals] and 5 [2.7%] had type 1 diabetes) [34], glucose‐lowering treatment (157 participants [85.7%] were receiving any glucose‐lowering agent, of which 32 [17.4%] were on insulin therapy), glycaemic control (91 [50.0%] participants showed an HbA1c < 7.0% [data available for 182 participants]) [35], and disease duration (68 [37.3%] participants had a diabetes duration ≥ 10 years [data available for 182 participants]) [36]. To calculate the incidence of T2DM, individuals without diabetes were followed from the time of assessment for study recruitment (November 2012–March 2015) until the date of the last blood test available when the computerised medical records were consulted (July 2022–August 2022).

Insulin resistance. Insulin resistance was estimated using the Homeostasis Model Assessment of Insulin Resistance (HOMA‐IR), which is fasting serum glucose (mg/dL) × fasting serum insulin (μU/mL) ÷ 405.23 [37]. Analyses regarding HOMA‐IR were conducted exclusively in participants not receiving insulin therapy (*n* = 1484), as the administration of exogenous insulin can confound the interpretation of HOMA‐IR [38].

### 2.3 Determination of Carbohydrate‐Deficient Transferrin

Serum CDT was determined using the commercial CAPILLARYS CDT kit on a MINICAP CDT device (Sebia, USA), as reported elsewhere [15]. The manufacturer's instructions were strictly followed. The assay is based on capillary electrophoresis. Transferrin glycoforms are separated by their electrophoretic mobility and electroosmotic flow in the capillary. The proteins are detected directly at the cathodic end of the capillary. During the analysis, serum transferrin is separated into 5 main glycoforms according to their degree of sialylation (asialotransferrin [non‐sialylated], disialotransferrin, trisialotransferrin, tetrasialotransferrin and pentasialotransferrin). Sialylation variants were observed in 17 individuals, and method interference was present in 22 individuals, preventing the determination of the percentage of 5‐, 4‐, and 3‐sialylated transferrin, but not CDT. Low sialylation transferrin glycoforms (i.e., disialotransferrin plus asialotransferrin) make up %CDT, which is automatically calculated by the system. The manufacturer recommends the following results interpretation: (1) normal (%CDT ≤ 1.3%), (2) indeterminate (%CDT > 1.3% and ≤ 1.6%) and (3) abnormal or indicative of alcohol abuse (%CDT > 1.6%). Total serum transferrin was measured using an ADVIA‐Centaur analyser (Siemens, Spain). Transferrin measurement was not available for 1 participant. Absolute CDT (in mg/dL) was estimated by multiplying the transferrin concentration by the %CDT.

### 2.4 Ethical Issues

The study was approved by the Regional Ethics Committee (code 2012–025) and adhered to the tenets of the Declaration of Helsinki. Written informed consent was obtained from all participants.

### 2.5 Statistical Analyses

The chi‐squared test (with test for trend when appropriate) was used to compare proportions. The Mann–Whitney test was used to compare numerical variables between groups, and the Jonckheere‐Terpstra trend test was used to compare numerical variables among ordinal categories. The Spearman's rank test was used to assess correlation. The generalised additive model for location, scale, and shape (GAMLSS) with beta distribution response was used for multivariate analysis of factors associated with %CDT. This method is well suited for response variables that are continuous and constrained within the (0,1) interval, such as proportions or rates, because it accounts for their non‐normal distribution and potential heteroskedasticity [39]. For ease of interpretation, beta regression coefficients were converted to odds ratios by exponentiating them. In this model, the first‐order interactions between the predictor variables and alcohol consumption were assessed. The inclusion of the interaction terms in the final model was based on a likelihood ratio test. To control the false discovery rate, the Benjamini–Hochberg correction was applied in the multivariate regression model including first‐order interactions. The association between %CDT and T2DM incidence was estimated using a proportional hazards Cox regression model. We estimated univariate Cox models, estimating the independent effect of CDT, as well as multivariate models, adjusting for the confounding effect of other clinical factors. The discriminatory power of the model was assessed using the C‐index. We also examined the association between the relative concentration of the various CDT glycoforms and the incidence of T2DM. These compositional data were incorporated into the prediction models as balances. A balance is a measure that represents a proportional relationship between subsets of parts of a composition, calculated as a linear combination of the logarithms of the parts weighted by coefficients reflecting their relative importance. First, observations with rounding zeros and missing values on some of the components were completed using a log‐ratio expectation maximisation imputation algorithm for compositional data [40]. Participants that presented missing values in all the composition were imputed using multiple chained equations [41], on the isomeric log‐ratio scale and backtransformed to the simplex space for further analysis. Second, we employed an elastic net model for censored data [42] to identify the balance with the highest predictive capacity for T2DM incidence. We conducted both a complete case analysis and a sensitivity, supplementary analysis based on the imputed dataset. The analyses were performed with the statistical analysis software R [43]. Specifically, the *gamlss* packages [44] were used to model the effect of clinical variables on CDT concentration. The *survival* package [45] was used to estimate Cox models. Lastly, the *coda4microbiome* package [46] allowed the relative concentration of glycoform variants to be treated as a composition.

## 3 Results

### 3.1 General Distribution of Serum Carbohydrate‐Deficient Transferrin Levels

**FIGURE 1:**
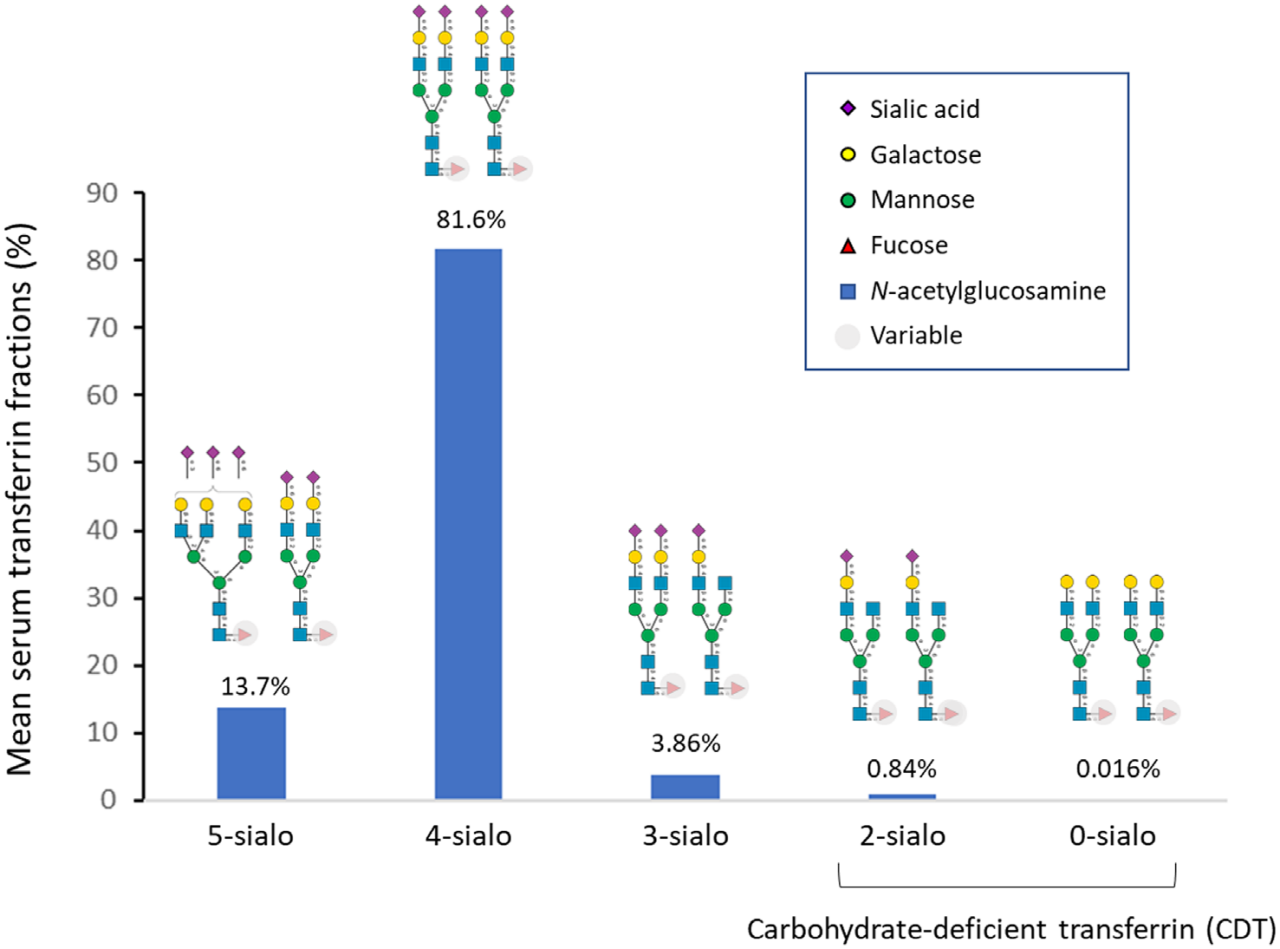
Schematic representation of serum transferrin *N*‐glycosylation and mean percentage of the various glycoforms (pentasialylated [5‐sialo], tetrasialylated [4‐sialo], trisialylated [3‐sialo], disialylated [2‐sialo], and asialylated [0‐sialo]) observed in the present study (*n* = 1477). The *N*‐glycans shown are only potential examples of the most frequent forms within the great variability in transferrin glycosylation [7, 12].

The distribution of the 5 transferrin glycoforms in the study population is shown in Figure 1. The most common glycoform was tetrasialylated. The median %CDT (i.e., the sum of disialylated and asialylated glycoforms) was 0.70% (mean 0.84%, interquartile range 0.50%–0.90%, range 0.10%–10.9%). The right‐skewed distribution (histogram) of CDT levels in percentages and in absolute terms (mg/dL) is shown in Figure S1.

### 3.2 Association of Carbohydrate‐Deficient Transferrin With Demographic and Lifestyle Variables

In the univariate analyses (Table 1), CDT concentrations (either as %CDT or in absolute terms) were higher in men than in women, and no significant association was observed with age or regular physical activity (Table 1). Serum CDT concentrations were higher in current smokers than in non‐smokers (Table 1). As expected, serum CDT concentrations were higher in alcohol drinkers, regardless of whether alcohol was measured in g/week or assessed using the AUDIT. Likewise, the effect of alcohol consumption on CDT was evident in light drinkers compared with abstainers (Table 1). There was also a significant trend for CDT to increase with increasing alcohol consumption (*p* < 0.001, trend test).

**TABLE 1:** Serum concentrations of CDT in relation to demographic, lifestyle, and metabolic factors.

Factor	CDT (%)			CDT (mg/dL)		
	No.	Median (IQR)	*p*	No.	Median (IQR)	*p*
Sex						
Woman (ref.)	838	0.6 (0.5–0.8)	—	837	1.7 (1.3–2.2)	—
Man	678	0.8 (0.6–1.0)	< 0.001	678	2.0 (1.4–2.7)	< 0.001
Age (years)						
18–35 (ref.)	296	0.7 (0.5–0.8)	—	296	1.8 (1.3–2.3)	—
> 35–50	413	0.7 (0.5–0.9)	0.179	412	1.8 (1.3–2.5)	0.661
> 50–65	391	0.7 (0.6–0.9)	0.010	391	1.8 (1.4–2.5)	0.280
> 65	416	0.7 (0.5–0.9)	0.415	416	1.7 (1.3–2.3)	0.399
Alcohol consumption (g/week)						
0–9 (ref.)	546	0.6 (0.5–0.7)	—	546	1.6 (1.2–2.1)	—
10–139	598	0.7 (0.5–0.8)	< 0.001	597	1.8 (1.3–2.3)	< 0.001
140–279	241	0.8 (0.6–1.1)	< 0.001	241	2.1 (1.6–2.8)	< 0.001
≥ 280	131	1.0 (0.8–1.8)	< 0.001	131	2.8 (2.0–4.7)	< 0.001
AUDIT (test points)						
0–7 (ref.)	1390	0.7 (0.5–0.8)	—	1389	1.8 (1.3–2.3)	—
≥ 8	126	0.9 (0.6–1.5)	< 0.001	126	2.5 (1.6–3.6)	< 0.001
Smoking (current)						
No (ref.)	1220	0.7 (0.5–0.8)	—	1219	1.7 (1.3–2.3)	—
Yes	296	0.8 (0.6–1.0)	< 0.001	296	2.0 (1.5–2.9)	< 0.001
Physical activity						
Low (ref.)	596	0.7 (0.5–0.9)	—	595	1.8 (1.4–2.4)	—
Moderate	552	0.7 (0.5–0.9)	0.324	552	1.8 (1.3–2.4)	0.288
High	368	0.7 (0.5–0.9)	0.760	368	1.8 (1.4–2.4)	0.726
Body mass index (kg/m^2^)						
≤ 25 (ref.)	424	0.6 (0.5–0.8)	—	424	1.7 (1.3–2.2)	—
25–30	574	0.7 (0.5–0.9)	0.019	573	1.7 (1.3–2.4)	0.160
> 30	518	0.7 (0.6–0.9)	< 0.001	518	1.9 (1.5–2.5)	< 0.001
Abdominal obesity^a^						
No	856	0.7 (0.5–0.9)	—	855	1.8 (1.3–2.4)	—
Yes	659	0.7 (0.6–0.9)	0.171	659	1.8 (1.4–2.4)	0.280
High blood pressure^a^						
No (ref.)	746	0.7 (0.5–0.8)	—	745	1.7 (1.3–2.3)	—
Yes	770	0.7 (0.6–0.9)	< 0.001	770	1.9 (1.4–2.6)	< 0.001
Low HDL‐cholesterol^a^						
No (ref.)	1249	0.7 (0.5–0.9)	—	1248	1.8 (1.3–2.5)	—
Yes	266	0.7 (0.6–0.8)	0.769	266	1.8 (1.4–2.2)	0.757
Hypertriglyceridemia^a^						
No (ref.)	1232	0.7 (0.5–0.8)	—	1231	1.8 (1.3–2.3)	—
Yes	277	0.7 (0.6–0.9)	0.001	277	1.9 (1.6–2.6)	< 0.001
Metabolic syndrome^a^						
No (ref.)	1202	0.7 (0.5–0.9)	—	1201	1.8 (1.4–2.4)	—
Yes	314	0.7 (0.6–0.9)	< 0.001	314	2.0 (1.6–2.5)	< 0.001
Insulin resistance^b^						
No (HOMA‐IR < 3, ref.)	980	0.7 (0.5–0.8)	—	979	1.7 (1.3–2.3)	—
Yes (HOMA‐IR ≥ 3)	504	0.7 (0.6–0.9)	< 0.001	504	2.0 (1.6–2.6)	< 0.001
Glycaemic status						
Normoglycaemia (ref.)	944	0.7 (0.5–0.8)	—	943	1.7 (1.3–2.3)	—
Prediabetes	389	0.7 (0.6–0.9)	0.004	389	1.9 (1.4–2.4)	0.024
Diabetes	183	0.7 (0.6–0.9)	0.002	183	2.0 (1.6–2.7)	< 0.001

Abbreviations: AUDIT, alcohol use disorders identification test; CDT, carbohydrate‐deficient transferrin; HDL, high‐density lipoprotein; HOMA‐IR, homeostatic model assessment for insulin resistance; IQR, interquartile range; Ref, reference category.

^a^ According to ATP‐III criteria [33].

^b^ Analyses regarding HOMA‐IR were conducted exclusively in participants not receiving insulin therapy.

### 3.3 Association of Carbohydrate‐Deficient Transferrin With Metabolic Variables

In univariate analyses (Table 1), individuals with obesity and those with metabolic syndrome had significantly higher CDT levels (either as %CDT or in absolute terms) than those without it, although the absolute differences were small (Table 1). Similarly, CDT levels were higher among individuals with some components of metabolic syndrome, such as hypertriglyceridemia and hypertension (Table 1). Among participants with hypertension (*n* = 770), CDT levels did not differ significantly according to antihypertensive treatment status (treated [*n* = 444] vs. untreated) or use of renin–angiotensin–aldosterone system inhibitors (yes [*n* = 382] vs. no) [47] (*p* > 0.05 for both comparisons; data not shown). Individuals with diabetes and prediabetes also had higher CDT levels than normoglycaemic individuals (Table 1). Among participants with diabetes, CDT levels did not differ significantly according to diabetes type (type 1 diabetes vs. T2DM), glycaemic control (HbA1c < 7% vs. ≥ 7%) [35], disease duration (diabetes duration ≥ 10 years vs. < 10 years) [36], use of glucose‐lowering agents (yes vs. no), or insulin treatment (yes vs. no) (*p* > 0.05 for all comparisons; data not shown). CDT levels were higher in participants with insulin resistance, as indicated by high HOMA‐IR levels (Table 1). There was a positive significant correlation between HOMA‐IR levels and %CDT (Rho = 0.133; *p* < 0.001).

**FIGURE 2:**
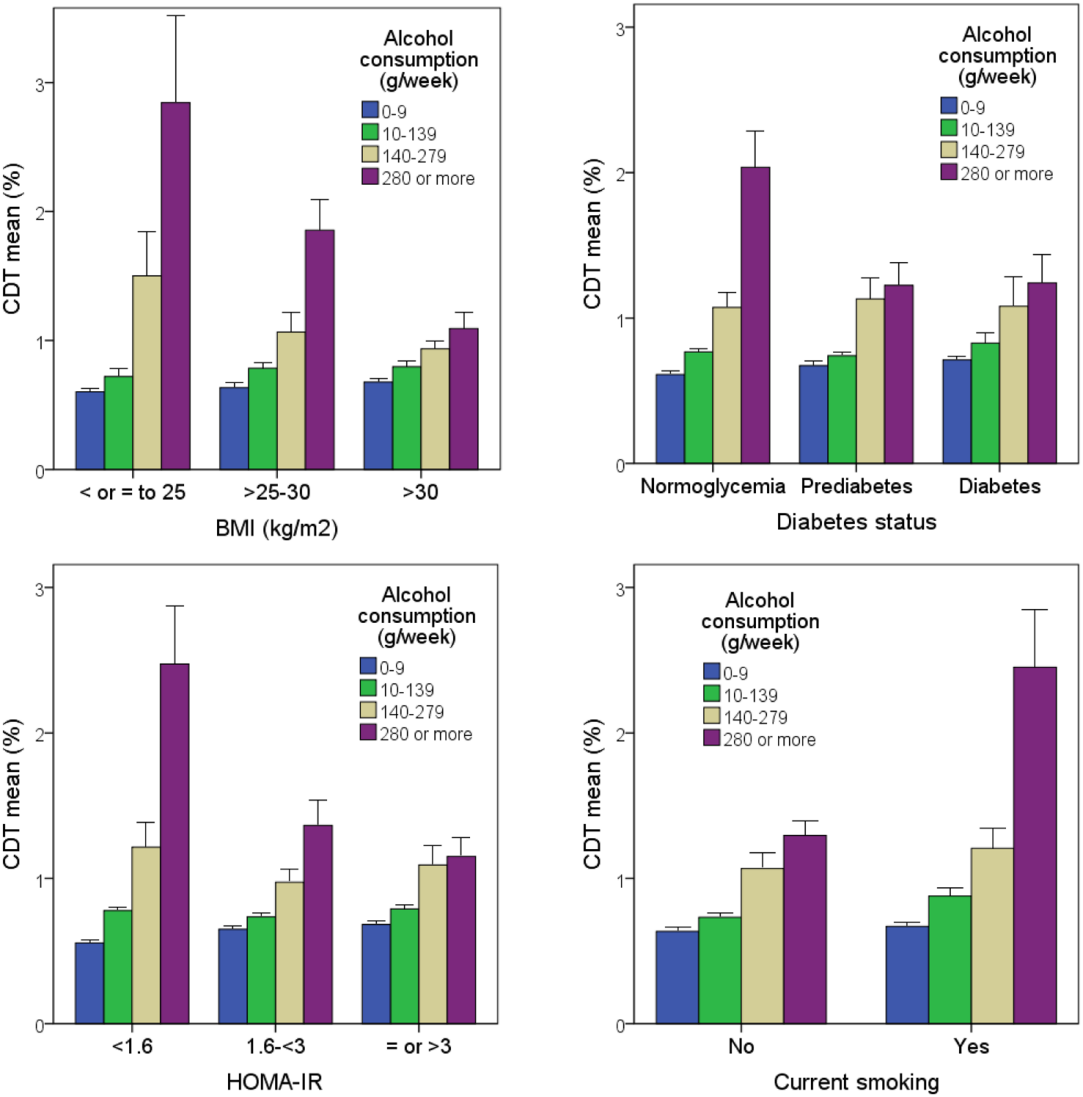
Mean CDT levels in the study population, stratified by alcohol consumption and metabolic variables (BMI, glycaemic status, and HOMA‐IR) and smoking. Calculations regarding HOMA‐IR were conducted exclusively in participants not receiving insulin therapy. Whiskers represent the standard error of the mean. CDT levels increase with alcohol consumption in all categories, but CDT levels in heavy drinkers (280 g/week or more) are lower with increasing BMI (*p* < 0.001), increasing insulin resistance (HOMA‐IR) (*p* < 0.001) and worsening glycaemic status (normoglycaemia < prediabetes < diabetes) (*p* = 0.017). In contrast, CDT levels in heavy drinkers are higher in smokers than in non‐smokers (*p* = 0.001).

Figure 2 shows the interaction (i.e., effect modification) between alcohol consumption and metabolic and lifestyle variables in relation to %CDT. The effect of alcohol consumption on %CDT was lower in participants with obesity and overweight than in those with normal weight. Similarly, the effect of alcohol consumption on %CDT was lower in participants with diabetes and prediabetes than in normoglycaemic individuals. Likewise, the effect of alcohol consumption on %CDT was lower in participants with insulin resistance (as estimated by HOMA‐IR) than in those without it. Conversely, the effect of alcohol consumption on %CDT was higher in smokers than in non‐smokers (Figure 2).

The multivariate analysis of factors associated with %CDT levels is shown in Table 2. As expected, alcohol consumption retained an independent association with %CDT levels. Conversely, male sex, hypertension, BMI, smoking, and diabetes per se were not significantly associated with %CDT levels after adjusting for confounders. Of note, smoking showed a positive significant interaction with heavy alcohol consumption (≥ 280 g/week) in relation to %CDT levels (Table 2), likely related to the observed higher alcohol intake among heavy‐drinking smokers compared to heavy‐drinking non‐smokers (Table 3). Conversely, obesity, prediabetes, and diabetes showed a negative significant interaction with alcohol consumption (especially with heavy drinking) in relation to %CDT values after adjusting for confounders (Table 2), despite similar alcohol consumption among heavy drinkers with and without diabetes or obesity (Table 3). Similar results were obtained when CDT was entered in mg/dL instead of %CDT in the multivariate models (data not shown). Table 3 also shows that the sensitivity of %CDT > 1.6% to detect heavy alcohol consumption decreases with increasing glycaemic status (from normoglycaemia to prediabetes and diabetes) and body mass index.

**TABLE 2:** Multivariate analysis of factors associated with %CDT.

Factor	OR	95% CI	*p*
Sex			
Woman (ref.)	1	—	
Man	1.01	(0.91–1.13)	0.864
Age (years)	1.00	(1.00–1.04)	0.103
Alcohol consumption (g/week)			
0–9 (ref.)	1	—	
10–139	1.08	(0.96–1.22)	0.316
140–279	1.41	(1.17–1.70)	0.002
≥ 280	1.89	(1.37–2.63)	0.001
Current smoking (yes)	1.11	(0.98–1.28)	0.202
High blood pressure (yes)			
No (ref.)	1	—	
Yes	0.98	(0.88–1.09)	0.864
Body mass index (BMI) (kg/m^2^)			
≤ 25 (ref.)	1	—	
> 25–30	0.98	(0.88–1.10)	0.889
> 30	1.06	(0.95–1.20)	0.406
Glycaemic status			
Normoglycaemia (ref.)	1	—	
Prediabetes	1.05	(0.94–1.17)	0.515
Diabetes	1.12	(0.97–1.29)	0.224
Interaction between sex and alcohol consumption			
Male and 10–139 g/week	1.14	(1.00–1.31)	0.116
Male and 140–279 g/week	1.21	(1.03–1.43)	0.070
Male and ≥ 280 g/week	1.28	(0.93–1.78)	0.224
Interaction between smoking and alcohol consumption			
Smoking and 10–139 g/week	0.98	(0.84–1.16)	0.904
Smoking and 140–279 g/week	1.03	(0.85–1.26)	0.864
Smoking and ≥ 280 g/week	1.36	(1.12–1.66)	0.009
Interaction between high blood pressure and alcohol consumption			
Hypertension and 10–139 g/week	1.02	(0.88–1.18)	0.864
Hypertension and 140–279 g/week	1.17	(0.99–1.39)	0.138
Hypertension and ≥ 280 g/week	1.21	(1.00–1.48)	0.116
Interaction between BMI and alcohol consumption			
25–30 kg/m^2^ and 10–139 g/week	1.04	(0.90–1.21)	0.732
25–30 kg/m^2^ and 140–279 g/week	0.81	(0.67–0.98)	0.095
25–30 kg/m^2^ and ≥ 280 g/week	0.90	(0.73–1.13)	0.526
> 30 kg/m^2^ and 10–139 g/week	1.00	(0.86–1.18)	0.951
> 30 kg/m^2^ and 140–279 g/week	0.69	(0.56–0.85)	0.003
> 30 kg/m^2^ and ≥ 280 g/week	0.58	(0.46–0.75)	< 0.001
Interaction between diabetes and alcohol consumption			
Prediabetes and 10–139 g/week	0.90	(0.78–1.05)	0.316
Prediabetes and 140–279 g/week	1.06	(0.90–1.26)	0.589
Prediabetes and ≥ 280 g/week	0.69	(0.57–0.85)	0.002
Diabetes and 10–139 g/week	0.85	(0.70–1.06)	0.253
Diabetes and 140–279 g/week	0.81	(0.65–1.02)	0.169
Diabetes and ≥ 280 g/week	0.71	(0.55–0.92)	0.037

*Note:* A generalised additive model for location, scale, and shape (GAMLSS) with beta distribution response was utilised because the response variable is continuous and constrained within the (0,1) interval. To facilitate interpretation, beta regression coefficients were transformed into odds ratios (OR, with 95% confidence intervals, CI) by exponentiating them. The model includes first‐order interactions with alcohol consumption. *p* values were adjusted using the Benjamini–Hochberg correction. Abbreviations: %CDT, carbohydrate‐deficient transferrin (expressed as a proportion); BMI, body mass index; Ref., reference category.

**TABLE 3:** Sensitivity of %CDT for the detection of heavy‐drinking according to glycaemic status, body mass index, and smoking status.

Factor	Alcohol consumption (g/week)	*p*	%CDT		*p*
			> 1.6% (true positive [sensitivity])	≤ 1.6% (false negative)	
Glycaemic status					
Normoglycaemia (*n* = 65)	350 (280–460)	0.246	25 (38.5)	40 (61.5)	0.022
Prediabetes (*n* = 44)	345 (280–400)		8 (18.2)	36 (81.8)	
Diabetes (*n* = 22)	360 (280–430)		4 (18.2)	18 (81.8)	
Body mass index					
≤ 25 kg/m^2^ (*n* = 18)	320 (280–420)	0.727	9 (50.0)	9 (50.0)	< 0.001
> 25–30 kg/m^2^ (*n* = 51)	350 (280–415)		19 (37.3)	32 (62.7)	
> 30 kg/m^2^ (*n* = 62)	350 (280–460)		9 (14.5)	53 (85.5)	
Smoking status					
Current smoker (*n* = 38)	420 (320–550)	< 0.001	17 (44.7)	21 (55.3)	0.007
Non‐smokers (*n* = 93)	320 (280–390)		20 (21.5)	73 (78.5)	

*Note:* Analyses include the 131 participants with heavy alcohol consumption (≥ 280 g/week). Data are expressed as median and interquartile range (within parentheses) or as absolute numbers and percentage (within parentheses). *p* values were obtained using the chi‐squared test (for categorial variables; the trend test was applied when there were > 2 ordinal categories), the Jonckheere‐Terpstra's trend test (for continuous variables, when there were > 2 ordinal categories), and the Mann–Whitney test (for continuous variables, when there were 2 independent categories). Abbreviation: %CDT, carbohydrate‐deficient transferrin (expressed as a proportion).

### 3.4 Predictive Value of Transferrin Glycoforms for Incident Type 2 Diabetes Mellitus

Among individuals without diabetes at baseline (*n* = 1333), relative quantification of the 5 transferrin glycoforms was unavailable for 33 individuals. Two participants were also excluded from these analyses because they developed diabetes secondary to pancreatic cancer during follow‐up. A total of 68 from the remaining 1298 individuals developed T2DM, with an incidence of 5.2% (95% CI, 4.1%–6.6%) after a median participant follow‐up of 7.4 years. After multiple imputation, the dataset included 1331 individuals, of whom 71 developed T2DM, corresponding to an incidence of 5.3% (95% CI, 4.2%–6.7%) over the same follow‐up period. As shown in Table 4 (complete case analysis) and Table S1 (sensitivity analysis based on multiple imputation for missing values), baseline %CDT concentration did not show a significant association with the T2DM incidence either univariately or after adjusting for additional clinical variables. The balance between pentasialotransferrin and trisialotransferrin tended to be associated with T2DM incidence in a univariate analysis, but this association was largely attenuated after adjusting for the effect of additional covariates (Table 4). In the same model, a higher BMI and prediabetes were significantly associated with incident T2DM (Table 4).

**TABLE 4:** Univariate and multivariate analysis (Cox regression) of factors associated with the incidence of type 2 diabetes mellitus.

	Univariate analyses		Multivariate analyses	
	HR (95% CI)	HR (95% CI)	HR (95% CI)	HR (95% CI)
Age (years)	—	—	0.99 (0.97–1.01) *p* = 0.203	0.99 (0.97–1.01) *p* = 0.236
Sex				
Woman (ref.)	—	—	1	1
Man			1.14 (0.66–1.97) *p* = 0.639	1.02 (0.58–1.80) *p* = 0.932
Body mass index (kg/m^2^)				
≤ 25 (ref.)	—	—	1	1
25 – < 30	—	—	1.58 (0.51–4.88) *p* = 0.425	1.53 (0.49–4.74) *p* = 0.465
≥ 30	—	—	4.28 (1.48–12.33) *p* = 0.007	3.97 (1.38–11.47) *p* = 0.011
Alcohol consumption (g/week)				
0–9 (ref.)		—	1	1
10–139	—	—	0.82 (0.46–1.48) *p* = 0.517	0.87 (0.47–1.61) *p* = 0.659
140–279	—	—	0.82 (0.41–1.65) *p* = 0.572	0.80 (0.39–1.62) *p* = 0.528
≥ 280	—	—	0.83 (0.33–2.10) *p* = 0.693	0.73 (0.27–1.98) *p* = 0.530
Current smoking				
No (ref.)	—	—	1	1
Yes			0.84 (0.34–2.04) *p* = 0.694	0.79 (0.32–1.91) *p* = 0.598
Prediabetes				
No (ref.)	—	—	1	1
Yes	—	—	17.3 (7.5–39.9) *P* < 0.001	16.1 (6.9–37.4) *P* < 0.001
CDT (%)^a^	0.75 (0.43–1.28) *p* = 0.334	—	0.51 (0.22–1.19) *p* = 0.118	—
Transferrin glycoform balance^b^	—	1.76 (0.94–3.28) *p* = 0.076	—	1.44 (0.72–2.87) *p* = 0.303
C‐index (95% CI)	0.47 (0.40–0.53)	0.58 (0.51–0.64)	0.86 (0.82–0.90)	0.86 (0.82–0.90)

Abbreviations: CDT, carbohydrate‐deficient transferrin; CI, confidence interval; C‐Index, Concordance Index; HR, hazard ratio; Ref, reference category.

^a^ Data available for 1331 individuals.

^b^ The transferrin glycoform balance with the highest predictive power was [log(pentasialylated transferrin) − log(trisialylated transferrin)]. Data available for 1298 individuals.

## 4 Discussion

In this general population‐based study, we observed that common metabolic disorders (obesity and diabetes mellitus) modify the effect of alcohol consumption on serum CDT levels, resulting in lower CDT levels among heavy drinkers. Although these metabolic disorders per se have little influence on CDT levels, their interaction with alcohol consumption is significant and could affect the diagnostic performance of CDT (particularly its sensitivity) as a marker of alcohol abuse. Thus, standard CDT thresholds may underestimate alcohol abuse in people with diabetes and/or obesity, potentially leading to underdiagnosis or misclassification.

To our knowledge, this is the first study in a general adult population to examine the interaction of alcohol consumption with BMI, glycaemic status, and insulin resistance (as estimated by the HOMA‐IR) in relation to CDT levels. According to our results, it appears plausible that a state of insulin resistance modifies the effect of alcohol consumption on transferrin sialylation. The mechanisms for this are not fully understood. Glycosylation abnormalities are a hallmark of diabetes [1, 48]. The enzymatic, active process of glycosylation (including sialylation) is distinct from the passive, non‐enzymatic glycation that is also a well‐known feature of diabetes, as evidenced by HbA1c. Sialic acids are involved in glucose transport and have been associated with reduced insulin sensitivity [49, 50]. Moreover, sialic acid concentrations have been found to be elevated in both obesity and diabetes [1]. Plausible mechanistic explanations for the increased sialic acids in patients with diabetes include increased desialylation by sialidases, stimulation of the hexosamine pathway and synthesis of acute phase proteins, and oxidative stress [1]. It is possible that the increased circulating levels of sialic acid observed in people with diabetes and/or obesity [1] lead to increased transferrin sialylation or interfere with the transferrin desialylation characteristic of heavy drinkers. However, this mechanism remains purely speculative and future studies are needed to evaluate whether increased sialic acid concentrations and their effect on transferrin sialylation could explain why insulin‐resistant heavy drinkers have lower CDT levels than non‐insulin‐resistant heavy drinkers.

Previous studies have suggested a J‐shaped association between alcohol consumption and insulin resistance [51], and consequently, with the risk of developing T2DM: light alcohol consumption has been associated with a lower risk of T2DM [52], whereas alcohol abuse has been linked to a higher risk [53]. Interestingly, other studies have also shown an interaction between alcohol consumption and BMI in relation to the development of T2DM, suggesting that the association between alcohol consumption and T2DM risk varies with BMI [54, 55]. In an attempt to disentangle the possible cross‐talk between changes in sialylation associated with alcohol consumption, BMI, and the development of T2DM, we examined the incidence of T2DM in this population after a median of 7 years. The main factors associated with the development of T2DM were, as expected, obesity and prediabetes status. However, alcohol consumption and CDT levels were not significantly associated with the development of T2DM in our study. We also investigated the possible association of other transferrin glycoforms with T2DM incidence, an aspect that has been little studied. In our experience, the balance between transferrin glycoforms was not significantly associated with the development of T2DM.

The main strengths of the study are its randomised design based on a general population with a wide age range and the availability of longitudinal follow‐up. In addition, the study included extensive phenotyping of lifestyle and metabolic variables. An additional strength is the consistency with previous studies in another study population in the same area, where similar results were observed regarding the interaction between alcohol consumption and BMI in relation to CDT levels (unpublished observation, Figure S2). The main weakness of the study is the lack of mechanistic assays to explain the interaction between alcohol consumption, insulin resistance, BMI, and diabetes in relation to CDT levels. The age and metabolic heterogeneity of the sample (an inherent feature of its population‐based design) made it necessary to employ multivariable analyses to control for the independent impact of each covariate on CDT levels. However, although the overall sample size was large, subgroup stratification (another valid approach to address potential confounding) resulted in relatively small sample sizes within each stratum. This may have limited the statistical power to detect significant differences when examining the impact of different metabolic characteristics on CDT levels among participants with diabetes (*n* = 183). Although CDT is a stable biomarker in individuals with consistent patterns of alcohol consumption [56, 57], the fact that it was only measured at baseline might still represent a limitation, given that changes in drinking behaviour over time could have influenced the long‐term prognostic value of both CDT and alcohol consumption in relation to the development of T2DM. Other objective alcohol biomarkers not measured in our study, such as phosphatidylethanol or urinary ethyl glucuronide [58], could have strengthened the assessment of alcohol intake beyond self‐reported data and improved the validation of CDT. Lastly, the racial homogeneity of the cohort, predominantly of White individuals from a single Spanish municipality, limits the generalisability of our results to populations of different ethnic backgrounds.

In conclusion, the results of the study have practical clinical implications. CDT values for the detection of alcohol abuse should be interpreted in light of the patient's BMI and glycaemic status, given that CDT might not be a sensitive marker for detecting alcohol abuse in people with obesity or diabetes. Further studies are needed to elucidate how alcohol consumption, glycosylation, and insulin resistance interact to influence CDT levels.

## Author Contributions

**Tomás González‐Vidal:** data curation (equal), visualization (equal), writing – original draft (equal). **Óscar Lado‐Baleato:** formal analysis (equal), methodology (equal), software (equal), validation (equal), visualization (equal). **Fátima de la Osa:** investigation (equal), writing – original draft (equal). **Manuela Alonso‐Sampedro:** data curation (equal), project administration (equal), validation (equal). **Carmen Fernández‐Merino:** investigation (equal), resources (equal), validation (equal). **Juan Sánchez‐Castro:** investigation (equal), validation (equal). **Francisco Gude:** funding acquisition (equal), methodology (equal), resources (equal), software (equal), supervision (equal). **Arturo González‐Quintela:** conceptualization (equal), data curation (equal), funding acquisition (equal), supervision (equal), visualization (equal), writing – original draft (equal).

## Conflicts of Interest

The authors declare no conflicts of interest.

## Supporting information


**Figure S1:** Histogram of CDT levels in percentage and in absolute terms (mg/dL).

**Figure S2:** Mean CDT concentrations in a validation study population (*n* = 413), stratified by alcohol consumption and body mass index. Whiskers represent the standard error of the mean. The adult participants were different from those in the present study although they were from the same health area, as described elsewhere (González‐Quintela A, et al. *Clin Exp Allergy*. 2003;33:199–205). For this validation study and as a sensitivity analysis, alcohol abusers were defined as women who habitually consumed more than 140 g of alcohol per week (equivalent to more than 20 g of alcohol per day) and men who habitually consumed more than 210 g of alcohol per week (equivalent to more than 30 g of alcohol per day). Light drinkers were defined as those individuals who habitually consumed alcohol but less than these amounts. Abstainers and occasional drinkers were included in the same group. According to BMI, individuals were considered to have normal weight (lean, < 25 kg/m^2^), overweight (25–30 kg/m^2^) or obesity (> 30 kg/m^2^). The method used to determine CDT was the same as in the main study. It was observed that BMI modifies the effect of alcohol consumption on CDT levels, with the effect being greater in lean individuals than in individuals with overweight or obesity.

**Table S1:** Univariate and multivariate analysis (Cox regression) of factors associated with the incidence of type 2 diabetes mellitus: sensitivity analysis using the imputed dataset (*n* = 1331).

## Data Availability

The data that support the findings of this study are available on request from the corresponding author. The data are not publicly available due to privacy or ethical restrictions.
