# Supplementary figures and images for: Diabetes and Obesity Modify the Effect of Alcohol Consumption on Carbohydrate‐Deficient Transferrin
Source: Endocrinol Diabetes Metab. 2025 Oct 23;8(6):e70112. doi: 10.1002/edm2.70112 (PMC12548559; doi:10.1002/edm2.70112)

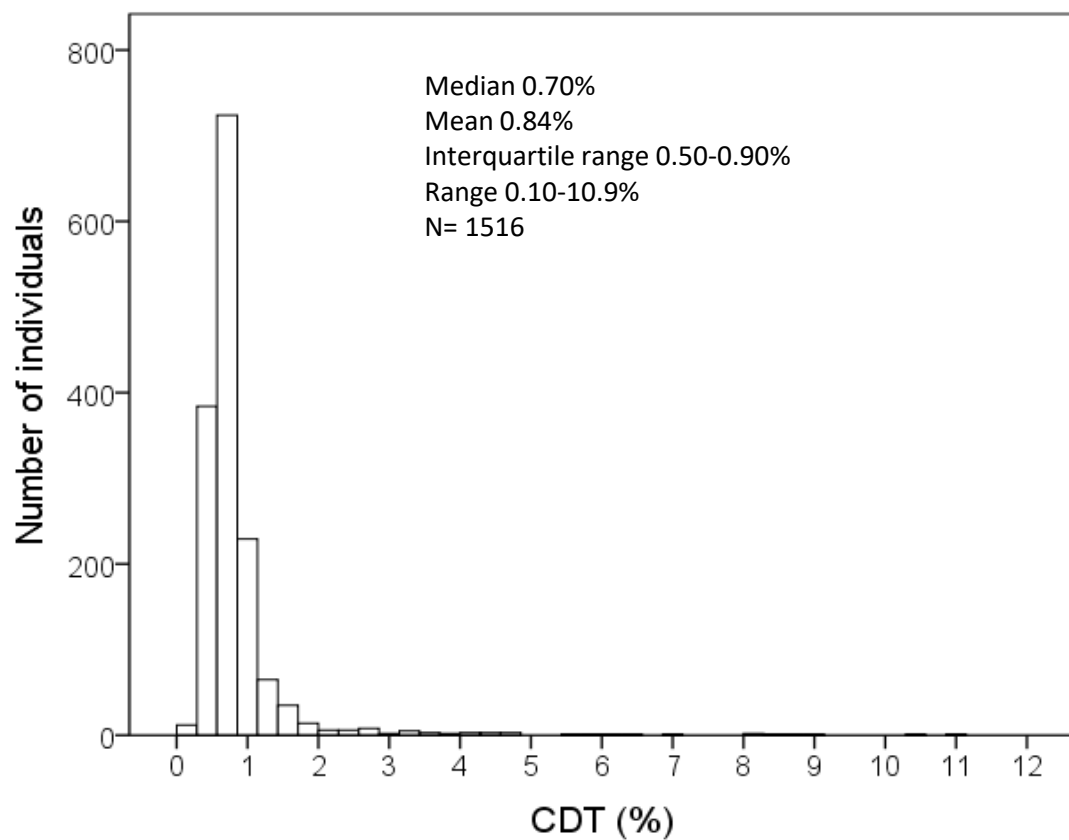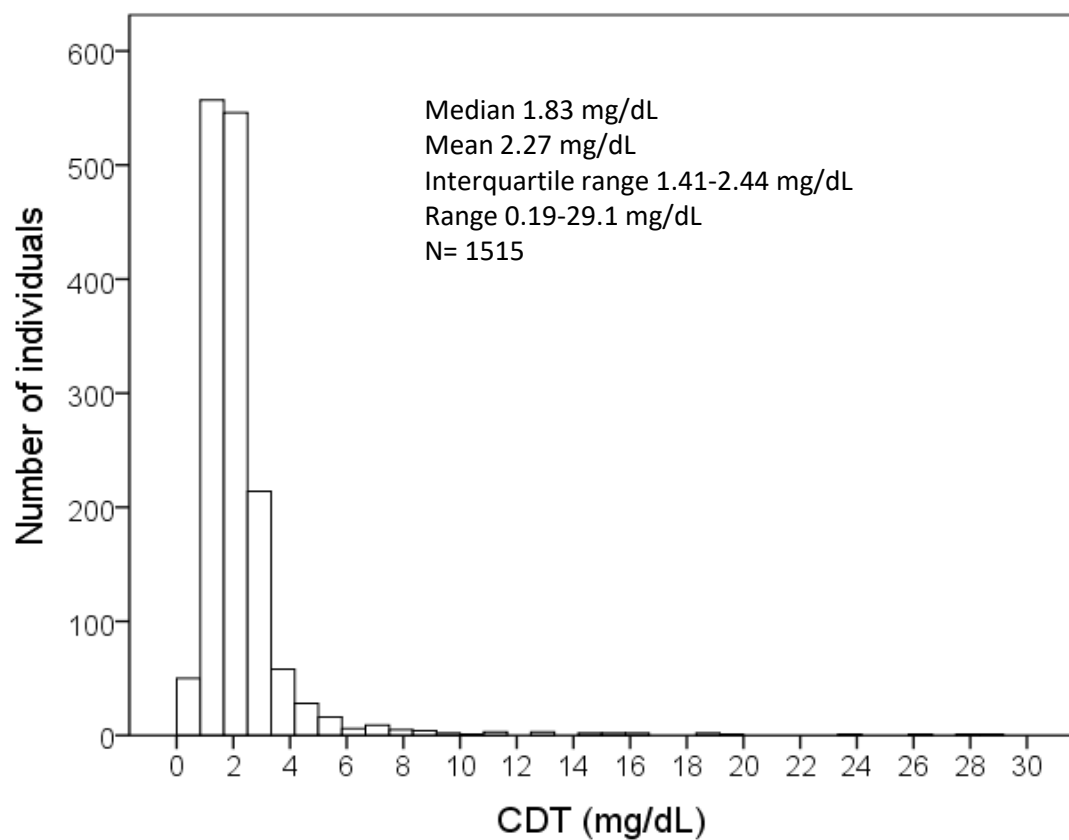

Supplement: Supplementary file 1 — Figure S1: Histogram of CDT levels in percentage and in absolute terms (mg/dL). [file EDM2-8-e70112-s002.pdf]

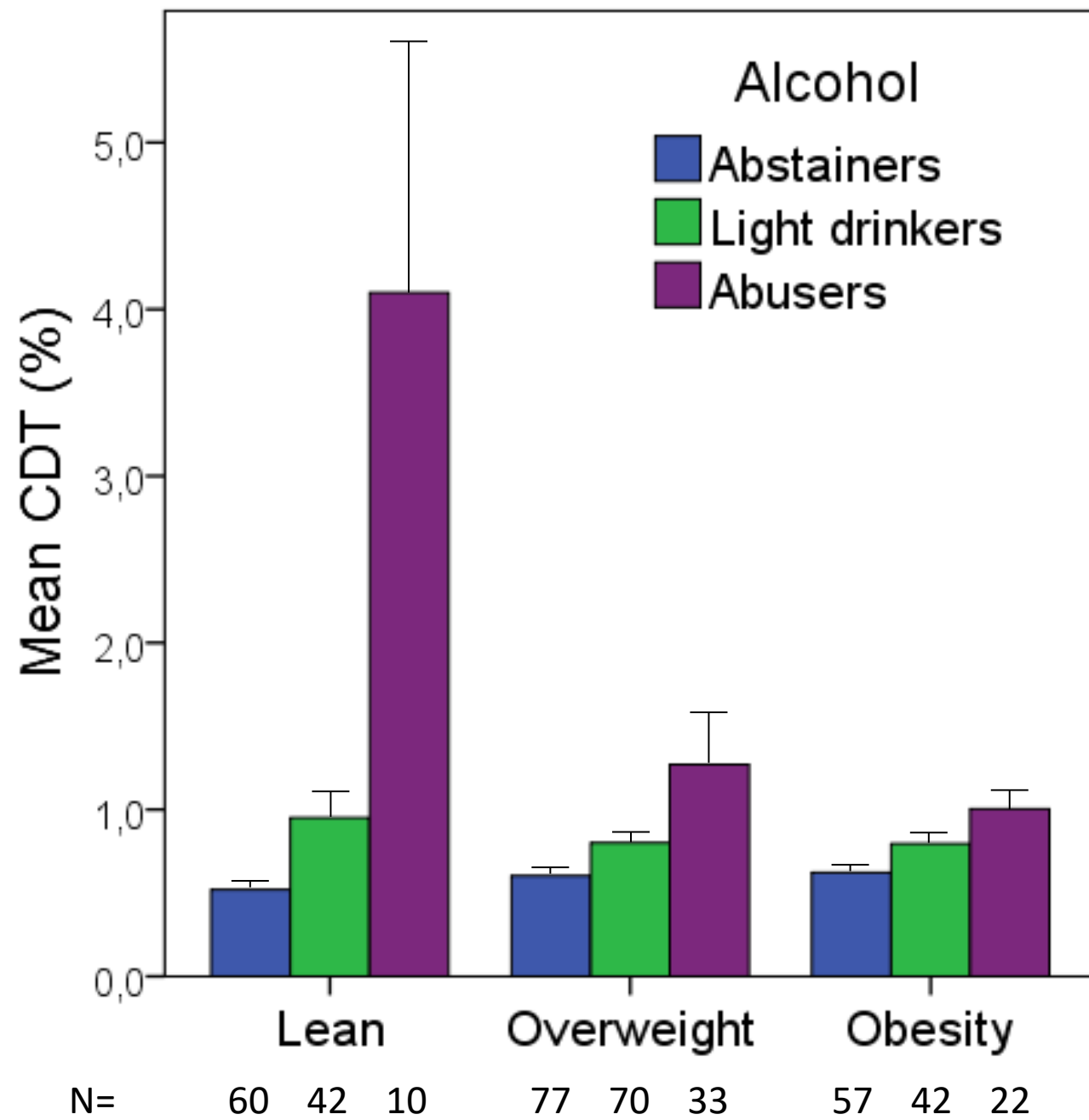

Supplement: Supplementary file 2 — Figure S2: Mean CDT concentrations in a validation study population (n = 413), stratified by alcohol consumption and body mass index. Whiskers represent the standard error of the mean. The adult participants were different from those in the present study although they were from the same health area, as described elsewhere (González‐Quintela A, et al. Clin Exp Allergy. 2003;33:199–205). For this validation study and as a sensitivity analysis, alcohol abusers were defined as women who habitually consumed more than 140 g of alcohol per week (equivalent to more than 20 g of alcohol per day) and men who habitually consumed more than 210 g of alcohol per week (equivalent to more than 30 g of alcohol per day). Light drinkers were defined as those individuals who habitually consumed alcohol but less than these amounts. Abstainers and occasional drinkers were included in the same group. According to BMI, individuals were considered to have normal weight (lean, < 25 kg/m2), overweight (25–30 kg/m2) or obesity (> 30 kg/m2). The method used to determine CDT was the same as in the main study. It was observed that BMI modifies the effect of alcohol consumption on CDT levels, with the effect being greater in lean individuals than in individuals with overweight or obesity. [file EDM2-8-e70112-s003.pdf]
