# Supplementary material for: Diabetes and Obesity Modify the Effect of Alcohol Consumption on Carbohydrate‐Deficient Transferrin
Source: Endocrinol Diabetes Metab. 2025 Oct 23;8(6):e70112. doi: 10.1002/edm2.70112 (PMC12548559; doi:10.1002/edm2.70112)
Supplement: Supplementary file 3 — Table S1: Univariate and multivariate analysis (Cox regression) of factors associated with the incidence of type 2 diabetes mellitus: sensitivity analysis using the imputed dataset (n = 1331). [file EDM2-8-e70112-s001.pdf]

Supplementary Table 1. Univariate and multivariate analysis (Cox regression) of factors associated with the incidence of type 2 diabetes mellitus: sensitivity analysis using the imputed dataset (n=1331)		
	**Univariate analysis**	**Multivariate analysis**
	**HR (95% CI)**	**HR (95% CI)**
**Age** (years)	--	0.99 (0.97-1.01)<br>P=0.231
**Sex**		
Woman	--	1<br>(reference)
Man		1.04 (0.60-1.80)<br>P=0.884
**Body mass index** (kg/m^2)		
≤25	--	1<br>(reference)
25 – <30	--	1.54 (0.50-4.76)<br>P=0.450
≥30	--	4.06 (1.41-11.69)<br>P=0.009
**Alcohol consumption** (g/week)		
0-9	--	1<br>(reference)
10-139	--	0.86 (0.47-1.55)<br>P=0.608
140-279	--	0.75 (0.37-1.50)<br>P=0.411
≥280	--	0.79 (0.31-1.81)<br>P=0.630
**Current smoking**		
No	--	1<br>(reference)
Yes		0.75 (0.31-1.81)<br>P=0.524
**Prediabetes**		
No	--	1<br>(reference)
Yes	--	16.8 (7.3-38.9)<br>P<0.001
**Transferrin isoform balance**[a]	1.76 (0.97-3.22)<br>P=0.065	1.46 (0.75-2.84)<br>P=0.271
**C-index** (95% CI)	0.58 (0.51-0.64)	0.86 (0.82-0.90)

[a]The transferrin isoform balance with the highest predictive power was [log (pentasialylated transferrin) – log (trisialylated transferrin)], as identified by a lasso regression model. HR, hazard ratio. CI, confidence interval. C-index, concordance index.
